# Meta-learning provides a robust framework to discern taxonomic carnivore agency from the analysis of tooth marks on bone: reassessing the role of felids as predators of *Homo habilis*

**DOI:** 10.1098/rsos.250548

**Published:** 2025-10-01

**Authors:** Manuel Domínguez-Rodrigo, Gabriel Cifuentes-Alcobendas, Marina Vegara-Riquelme, Edgard Camarós, Enrique Baquedano

**Affiliations:** ^1^ Department of Anthropology, Rice University, Houston, TX, USA; ^2^ Institute of Evolution in Africa, Archaeological and Paleontological Museum of Madrid & Rice University, Alcalá de Henares, Madrid, Spain; ^3^ Department of History and Philosophy (Area of Prehistory), Alcalá University, Alcalá de Henares, Madrid, Spain; ^4^ Area of Prehistory, History Department, University of Santiago de Compostela and CISPAC, Praza da Universidade 1, Santiago de Compostela, Spain; ^5^ Archeological and Paleontological Museum of Madrid, Alcalá de Henares, Madrid, Spain

**Keywords:** taphonomy, computer vision, tooth marks

## Abstract

Determining carnivore agency in taphonomic research is crucial for identifying site formation processes and carnivore–hominin interactions that influenced human evolution. Previous deep learning (DL) models classified the four principal carnivore agents affecting African hominins, but exhibited uneven performance due to unbalanced sample sizes. This study introduces a dual method based on few-shot supervised learning (FSSL) and model-agnostic meta-learning (MAML) as an alternative, achieving more consistent accuracy (FSSL: 81.54–83.56%; MAML: 82.56–85.13%), and significantly improving macro-average F1 scores. The best performing MAML model, Xception, reached 85.13% accuracy and an 84% F1 score, with taxon-specific F1 scores of 82% (crocodiles), 83% (hyenas), 88% (leopards) and 83% (lions), making the most precise classification of carnivore-made tooth marks to date. Applying FSSL-MAML ensemble models to *Homo habilis* specimens OH7 and OH65 from Olduvai Gorge confirms that leopards were preying on these hominins, as they had been earlier on australopithecines. Contrary to our expectations, these findings demonstrate that early *Homo* was still part of the prey spectrum, reinforcing the idea that the transition to dominant predator status occurred later in human evolution or penecontemporaneously to *H. habilis* through a different hominin taxon.

## 1. Introduction

The taxon-specific identification of carnivore agency has become a cornerstone in archaeological reconstructions of hominin–carnivore interactions. Understanding which carnivores contributed to bone accumulations provides essential insights into trophic dynamics, behavioural ecology and the selective pressures that shaped hominin evolution. Felids (namely lions, leopards and sabertooth cats) have been hominin predators in the distant past, and have been argued to have provided kleptoparasitic opportunities for our ancestors [1–5] (but see [6,7]). Crocodiles have also been posited as potential hazards for hominins and as providers of animal carcasses through opportunistic behaviours [8,9] (but see [10,11]). The hunting-and-scavenging debate in the origin of the genus *Homo* and in the emergence of the archaeological record hinges on detecting a primary taphonomic signal by felids or hyenids in archaeofaunal assemblages [12–16]. Given the ecological significance of lions, leopards, hyenas and crocodiles as both potential competitors and predators of early hominins, taxon-specific identification of their taphonomic signatures has become essential. Testing all the hypotheses that involve hominin–carnivore interactions requires robust identification of carnivore-specific bone modifications, as different carnivores impose distinctive taphonomic signatures that can reveal patterns of primary and secondary carcass access by hominins.

Recent advancements in geometric morphometrics (GMM) and machine learning (ML) have potentially improved our ability to distinguish taxon-specific carnivore signatures [11,17–19]. GMM analyses of tooth marks have demonstrated high classification accuracy; however, these studies have been limited by small sample sizes, which may bias results toward larger, more distinct tooth marks, while under-representing smaller and potentially ambiguous marks [20]. Expanding these datasets is crucial for refining statistical models and capturing the full range of variation in carnivore-made bone surface modifications (BSM). These methods have also oversimplified tooth mark morphologies by making classifications on tooth pit shapes that either have not been documented in specific carnivore taxa or are based on only a fraction of all the tooth pit morphologies that those carnivores can carry out [20]. This invalidates statements about general taxonomic discrimination, since the taxon-specific morphological spectrum is also under-represented in those studies.

With the above-described goals in mind, a pilot study on taxonomic agency detection was carried out using deep learning (DL) computer vision (CV) methods [21], as a complement to the available GMM studies. However, CV approaches have demonstrated significant potential for taxon-specific carnivore identification [22–25]. DL models trained on standardized bidimensional images of tooth marks (both pits and scores) achieved promising results in differentiating multiple carnivore (lion, leopard, hyena and crocodile) taxa [21]. A prior study comparing five different carnivore species achieved an initial classification accuracy of 56%, which improved to over 70% after refining image standardization techniques [26]. The CV study showed a higher but variable accuracy, depending on the DL model used [21]. A ResNet50 transfer learning (TL) model yielded 88% accuracy. DenseNet201 resulted in 84.3% accuracy on the same dataset. The other two models (VGG19 and EfficientNet B7) ended up with poorer performance (accuracy = 80%) [21]. Given the unbalanced nature of the datasets, with a small sample representing crocodiles and only a moderately bigger one for leopards, accuracy is not the adequate metric to certify the performance of those models. The macro-average F1 score for the highest achieving model (ResNet50) was 81. The F1 score is a metric used in classification tasks to assess a model’s performance by balancing precision and recall. Precision is a way to assess the proportion of false positives. For example, the precision for crocodiles was 0.90, meaning that 90% of the time when the model predicted ‘crocodile’, it was correct. Recall (also known as sensitivity or true positive rate) measures how many actual positive cases were correctly identified by the model. The recall for crocodiles was 0.45, meaning that out of all actual crocodile cases, the model correctly identified only 45%. This suggests that the model is missing many crocodile instances (high false negatives). The F1 scores for the other models were even more unbalanced. In essence, the four models seemed competent to identify tooth marks from leopards, hyenas and lions (in that order, which is reflecting their proportional sample sizes), but they were inefficient at identifying crocodile tooth marks. In addition, the small sample sizes for the four carnivores only allowed us to use a training-validation split of the datasets if we were to achieve any resolution using data-hungry DL models. We had to renounce the use of hold-out testing sets, thereby not being secure about the generalization properties of the resulting models [27].

These two reasons together compelled us to use an alternative method of CV that could be efficient at capturing class properties using small datasets and also at being able to learn and use hold-out testing sets without compromising the learning process. This alternative method is the reason why we repeat the analysis of the four datasets here, implementing a meta-learning analysis to compare with the original DL analysis. The main goal of this meta-learning analysis here is not just to produce models with high accuracy (and presumably, low loss), but models that show a balance between precision and recall, where the target is high macro-average F1 scores (greater than 80%), in which individual carnivore F1 scores are at least 80% or more.

Meta-learning, often described as ‘learning to learn,’ is an ML approach designed to enhance a model’s ability to quickly adapt to new tasks with minimal data [28–32]. It is commonly referred to as adaptive artificial intelligence (AI), because it adapts to datasets instead of having to adapt datasets to the method, as is the case for traditional DL. Unlike traditional DL models that require vast amounts of training data for each specific task, meta-learning focuses on developing generalizable learning strategies that allow models to efficiently solve new problems with only a few examples and a large array of classes compared at the same time. At its core, meta-learning operates at two levels: the inner and the outer levels. The inner level refers to task-specific learning, where the model attempts to learn a single task, such as classifying BSM. The outer level, on the other hand, optimizes the learning strategy across multiple tasks, ensuring that the model can generalize and quickly adapt to new situations. This approach is commonly used in few-shot learning, where models must perform well despite having access to only a handful of labelled examples. One of the most widely known meta-learning techniques is model-agnostic meta-learning (MAML), which optimizes a model’s parameters so that it can rapidly adjust to a new task with just a few gradient updates. The properties of MAML make it one of the most suitable methods for taphonomic datasets, where samples are commonly fairly reduced and unbalanced. Inspired by meta-learning few-shot methods, we also implement here a hybrid approach based on readapting a meta-learning framework to a supervised learning structure, creating a few-shot supervised learning (FSSL) model that enables the use of triple splitting of data (training–validation–testing) in the same way as other meta-learning procedures.

Here, we will reanalyse the four carnivore samples published by Domínguez-Rodrigo *et al*. [21] using few-shot methods through FSSL-MAML modelling. We will provide robust models of carnivore agency identification and more secure referential framework for interpreting carnivore agency in archaeological and palaeontological palimpsests, where the hominin–carnivore interaction and/or those of various carnivores is the main target in the analysis of BSM.

## 2. Sample and method

### 2.1. Sample

A total of 1296 tooth marks, including both pits and scores, were analysed (table 1). This corresponds to the original dataset used by [21], plus some additional marks from crocodiles described in [33,34]. This study aimed to establish a robust reference image database for the four most common extant carnivores hypothesized to have interacted with hominins in the African past: lions (*Panthera leo*), leopards (*Panthera pardus*), hyenas (*Crocuta crocuta/Hyaena hyaena*) and crocodiles (*Crocodylus* spp.). The dataset comprises tooth mark samples distributed as follows: lions (*n* = 264), leopards (*n* = 544), hyenas (*n* = 364) and crocodiles (*n* = 124) (table 1).

**Table 1.** Main characteristics of the tooth mark experiments for the four carnivores (crocodiles, hyenas, leopards and lions) [21].

carnivore	scientific name	number and age	**weight (kg)**	diet	bones used	consumption details	cleaning method
hyena	*Hyaena hyaena*	4 individuals: 16F, 18M, 8F, 6F	30, 35, 32, 28	3 kg of lean meat with bone (two days/week), fruit (other days)	27 femurs, 6 humeri, 9 radii, 14 tibias	each individual consumed bones separately. Bones collected after 1 h to maximize recovery.	neutral detergent and boiling water
lion	*Panthera leo persica*	3 individuals: 12M, 8F, 8F	198 (M), 150−160 (F)	6 kg of lean meat/day (M), 6 kg shared between females, 1 day fasting	16 bovine limbs (8 front, 4 hind)	bones collected after 12 h. Males and females fed separately. Zookeepers documented process.	neutral detergent and boiling water (1.5 h drying)
leopard	*Panthera pardus saxicolor*	3 individuals: 9M, 4M, 4M	70 (M), 60 (young males)	adjusted to usual diet	12 adult sheep limbs	young leopards consumed 8 limbs, adult consumed 4. Bones boiled for 6 h, submerged in hydrogen peroxide for 24 h.	water and hydrogen peroxide for 24 h
crocodile	*Crocodylus niloticus* plus *Osteolaemus tetraspis*	8 females: 1 small (1.3 m), 2 medium (1.8 m), 5 large (2.3 −3.1 m) for *Crocodylus niloticus*.	not specified	fed once a week for 4 months for *Crocodylus niloticus*	19 partial carcasses (suid and bovid limbs) for *Crocodylus niloticus*	carcasses monitored for 1.5 h, collected after 15 h of exposure for *Crocodylus niloticus* and after 10 h for *Osteolaemus tetraspis*	boiling in water and hydrogen peroxide for 24 h for *Crocodylus niloticus*

Previous analyses of bidimensional tooth marks used an Optika trinocular microscope (SZM-1), which exhibited limitations in depth of field, resulting in unfocused areas in several images. To enhance image resolution and clarity, a new database was generated using a Leica Emspira three digital microscope, which captures both bidimensional and tridimensional images. This microscope employs image stacking techniques to eliminate unfocused regions by merging multiple overlapping images of the same mark. In addition, its 12 megapixel sensor provides a sharper image than the previous equipment used. Unlike prior studies, we used colour imaging [20] and primarily employed ×30/32 magnification for tooth scores, with the magnification for tooth pits variable, ranging from ×7.5 to ×60. In cases where tooth marks were exceptionally small, magnification was increased to ×60. Additionally, whereas previous studies analysed tooth pits and scores separately, we opted to aggregate them into a single category. This approach minimizes ambiguity in distinguishing between pits and scores, mitigates potential classification inconsistencies due to overlapping boundaries and enhances the modelling process by increasing the dataset available for algorithmic learning.

Furthermore, most of the experimental sample consisted of newly acquired data initially documented in [21]. Carcasses from small (goat, sheep, boar and pig), medium-sized (deer) and large (cattle) ungulates were used in the feeding experiments, as detailed in table 1.

Image standardization was performed using bidimensional matrices for centring and normalization, employing the preprocessing functions specific to each architecture. All the images were resized to the original size of the images used for the TL models. This resulted in resizing of images at 224 × 224 pixels for the ResNet and DenseNet models and 299 × 299 pixels for the Xception and the EfficientNet V2L models. Given that the most tooth marks appear highly similar to the human-trained eye, this high-definition approach was intended to facilitate the DL process in identifying classificatory differences. Samples were divided into training (70%), validation (15%) and testing (15%) sets. This resulted in 907 images for training (sampling the four carnivores) and 390 images split between validation and testing.

The deep convolutional neural network (DCNN) models were implemented using the Keras (v. 2.4.3) application programming interface (API) with a TensorFlow (v. 2.3.0) back end. Computation was conducted on a HP Zbook Studio G10 workstation with 64 GB RAM, within a CUDA-based cuDNN computing environment. All code was written in Python 3.7.

### 2.2. Few-shot methods implemented

Given the sample size and its unbalanced nature in this study, a special consideration was taken to minimize the risk of validation overfitting observed in previous modelling efforts. To achieve this, we employed an independent testing set evaluated through two distinct DL strategies within the few-shot learning (FSL) paradigm. The first approach was an FSSL method, while the second was an MAML algorithm, both of which are designed to enable effective model training when only limited labelled data are available. The first approach, FSSL, was conceptually inspired by *multi-task learning* (MTL)-based few-shot techniques, although it does not qualify as a meta-learning method. It adopts an *n-way k-shot* formulation, where multiple mini-batches are drawn for each class and model optimization is carried out under a supervised TL scheme. This set-up leverages pre-trained feature extractors while fine-tuning on the small target dataset, enhancing discrimination capability with minimal data.

The second approach, MAML, differs fundamentally in its training procedure. MAML incorporates an episodic learning process, in which the model is trained over a series of tasks using a *support set* for parameter initialization and a *query set* for evaluation. The method involves an *inner-loop* task-specific adaptation phase, followed by a *meta-gradient* update in the *outer loop*, enabling the model to learn parameter initializations that are highly adaptable across diverse tasks. This flexibility allows the model to rapidly adapt to unseen classes with minimal fine-tuning.

### 2.3. Model architecture

The FSSL-MAML model employed in this study was designed within a TL framework, using pre-trained models as feature extractors. The selected base architectures included both classical and modern DL frameworks, chosen based on their demonstrated success in previous taphonomic classification tasks. These models comprised ResNet50 and DenseNet201, recognized for their robust feature extraction capabilities, alongside more advanced architectures such as ResNet152, Xception and EfficientNetV2L. The latter architectures incorporate sequential and non-sequential network designs, integrating sophisticated computational strategies to enhance performance in classification tasks. Their effectiveness has been previously validated in palaeontological and taphonomic research, and more recently, in actualistic modelling.

The architectures used in this study can be classified into four distinct types based on their structural characteristics:

(1) *Residual networks (ResNet family)*. These architectures employ skip (residual) connections, which facilitate gradient propagation and enable the training of very deep networks without vanishing gradient issues.
(2) *Densely connected networks (DenseNet201)*. This approach enhances computational efficiency and gradient flow by linking each layer to every preceding layer, thereby improving feature reuse.
(3) *Depthwise separable convolutional networks (Xception-based architectures)*. This category, originating from the Inception architecture, replaces standard convolutional layers with depthwise separable convolutions, optimizing computational efficiency while maintaining performance.
(4) *Compound scaled efficient networks (EfficientNet V2L)*. These architectures implement compound scaling, optimizing network depth, width and resolution simultaneously. EfficientNetV2L models integrate fused convolutional layers and advanced regularization techniques, providing an optimal trade-off between computational cost and accuracy.

### 2.4. Integration of few-shot supervised learning and model-agnostic meta-learning with pre-trained (transfer learning) models

TL offers several advantages, particularly in scenarios with limited training data, such as FSL. By leveraging pre-trained models, TL enables the reuse of feature representations learnt from large datasets, reducing the need for extensive labelled data and significantly accelerating training. This approach enhances model generalization by incorporating robust, high-level features extracted from diverse domains, which can be fine-tuned for specific tasks. Additionally, TL mitigates overfitting by reducing the number of trainable parameters, thereby improving performance on small datasets. It also lowers computational costs by using pre-trained feature maps instead of training deep networks from scratch. In meta-learning contexts, such as MAML-based architectures, TL further enhances adaptability, allowing models to quickly adjust to new classification tasks with minimal updates.

The meta-learning approach employed in this study aimed, therefore, to leverage the advantages of pre-trained feature extractors while optimizing the classification of carnivore samples. The FSSL-MAML-specific models were constructed atop the output feature maps of the last convolutional layer from each pre-trained base model. Subsequent layers were added to enhance feature representation and classification performance. The initial layers of the FSSL-MAML models consisted of two residual blocks, each incorporating depthwise separable convolutional layers (kernel size = 3, stride = 1 and padding = ‘same’). These layers facilitated hierarchical feature learning, an essential component of FSL, where nuanced patterns must be extracted from limited samples. The reduction in computational cost was achieved through depthwise separable convolutions, which decompose standard convolutions into spatial and depthwise operations, reducing the number of trainable parameters.

### 2.5. Attention mechanisms for feature enhancement

To enhance feature detection, we integrated squeeze-and-excitation (SE) attention mechanisms within the residual blocks. SE blocks dynamically recalibrate channel-specific features, enabling the model to prioritize the most informative elements of the input data. SE attention serves as a self-attention mechanism, refining feature selection as input data propagates through successive convolutional layers. Following the residual blocks, we incorporated a global average pooling (GAP) layer, which compressed spatial information while retaining critical channel-specific features. GAP prevents overfitting by reducing the number of parameters, ensuring a more generalizable feature representation. This layer was followed by a 512-neuron dense layer with ReLU activation, further refining feature extraction.

### 2.6. Regularization strategies and optimization techniques

To mitigate overfitting, we incorporated multiple regularization techniques:

(1) *Dropout*. We tested dropout rates ranging from 0.3 to 0.8, but observed minimal variation in performance. This is probably due to the interaction between dropout and batch normalization (BN), which stabilized learning by normalizing feature distributions.
(2) *Batch normalization*. BN counteracted the potential drawbacks of dropout by scaling and shifting feature distributions, ensuring stable training dynamics.
(3) *Early stopping*. We monitored validation loss, halting training if no improvement was observed after 15 epochs. The model was restored to the best performing weights.
(4) *Learning rate scheduling*. A progressive learning rate reduction strategy was implemented, decreasing the learning rate by a factor of 0.1 at predefined epochs to enhance model convergence.

Additionally, data augmentation was employed to increase sample diversity. The augmentation pipeline included (as specified in [33]): random rotation (±20°), width and height shifts (0.2 of image size), horizontal flipping. In addition to colour normalization, a specialized protocol was subsequently implemented to assess potential biases arising from colour variations within the tooth mark dataset. To achieve this, we developed a function to perform colour-based data augmentation, adhering to the protocol established by Cifuentes-Alcobendas [35]. This function introduces controlled variations in hue, saturation and brightness within the hue, saturation, value (HSV) colour space, enhancing the model’s robustness against differences in lighting and colour conditions. The process begins by converting images from the red, green, blue (RGB) colour space to the HSV colour space, which facilitates precise tone modifications. Hue (H) represents the fundamental colour type (e.g. red, green, blue) on a circular scale ranging from 0° to 180°. The implemented algorithm applies random adjustments to the red–green–blue balance, thereby shifting the original colour along these three channels. Saturation (S) governs the intensity of the colour, determining whether it appears more vivid or muted. This modification is particularly useful for datasets sourced from varying imaging equipment. Finally, value (V) represents the brightness level, controlling whether a specific colour appears darker or lighter. Adjustments in brightness contribute to the convolutional neural network’s (CNN) ability to handle images with varying contrast levels.

The HSV-based augmentation process independently modifies hue, saturation or brightness for each augmented image. The randomization mechanism selects one of these three attributes for adjustment. If hue is selected, a random value between −10° and 10° is added to the hue channel. If saturation is adjusted, the saturation channel is scaled by a factor randomly chosen between 0.8 (decreasing saturation) and 1.2 (increasing saturation). In the case of brightness modification, the value channel is scaled within the same range (0.8–1.2), thereby making the image darker or lighter. These modifications are carefully constrained to prevent extreme alterations that could negatively impact CNN performance. Following the augmentation process, each image is converted back from HSV to RGB to ensure compatibility with CNN processing requirements. Subsequently, colour normalization is applied and the image is rescaled to fit the input dimensions required by the network. This comprehensive approach mitigates potential biases introduced by colour differences in the dataset while improving the model’s generalization to diverse imaging conditions.

In addition to regularization methods, data and colour augmentation protocols, we also experimented with optimization variation. There were three optimizers used: Adam, Adagrad and Stochastic Gradient Descent (SGD). SGD is an optimization algorithm that updates model parameters using a randomly selected subset (mini-batch) of the training data rather than the entire dataset. This reduces computation time and helps escape local minima by introducing noise in the updates. However, SGD suffers from issues such as slow convergence, difficulty in handling sparse data and the need for careful tuning of the learning rate. To address these limitations, variants like Momentum and Nesterov Accelerated Gradient (NAG) (not used here) have been developed to improve the efficiency of SGD.

Adagrad (Adaptive Gradient Algorithm) enhances SGD by adapting the learning rate for each parameter based on past gradients. It assigns smaller learning rates to frequently updated parameters and larger learning rates to infrequently updated ones. However, Adagrad’s main drawback is that its learning rate continuously decreases over time, which can slow down training and cause the algorithm to stop learning prematurely. A good intermediate solution is provided by the Adam (Adaptive Moment Estimation) optimizer. Adam combines the advantages of both SGD with Momentum and Adagrad. It maintains two moving averages for each parameter: one for the gradients (first moment) and another for the squared gradients (second moment). These moving averages help Adam adjust the learning rate dynamically, making it more robust to noise and capable of handling sparse data effectively. Adam generally converges faster than both SGD and Adagrad and is widely used in DL applications, particularly in training deep neural networks. For MAML algorithms, it is commonly one of the best performing optimizers, providing more stable and accurate models [20,27].

### 2.7. Baseline model for comparative evaluation

While complex models can yield superior results, simpler architectures often perform comparably in specific tasks. To assess the performance differential, we constructed a baseline model, retaining identical regularization, augmentation and optimization techniques while reducing architectural complexity. The baseline consisted of: (i) a single two-dimensional convolutional layer (512 filters), (ii) a GAP layer, (iii) a 512-neuron dense layer, (iv) dropout + batch normalization layers and (v) a final dense layer with softmax activation. The performance of both FSSL-MAML architectures (complex versus baseline) was compared across all agency classifications of the four carnivore samples and the best architecture (as reflected in highest accuracy plus lowest loss) was selected for each TL model used.

### 2.8. Shot-task configurations in few-shot learning

FSL models require careful tuning of shot-task configurations. Shot refers to the number of labelled samples per class. Task represents a single classification episode involving different data subsets. The balanced combinations of shots and tasks produce optimal results, but such a shot-task proportion is not easy to achieve. For this reason, we tested two configurations:

(A) *Low-shot scenario*. Fewer labelled samples per class, but an increased number of tasks. For FSSL, this involved using a 5-shot to 40-task ratio. For MAML, this was performed using a 5-shot to 40-task ratio with a three query process, to keep the number of classes (n_way), examples per class (k_shots), number of tasks (n_tasks, inner loop) and evaluation episodes (q_query, outer loop) in proportion to the image sample size, in order to avoid any instance of overfitting.
(B) *High-shot scenario*. A larger number of labelled samples per class, with fewer total tasks. Here, this involved the use of a 10 shot to 20 task ratio for FSSL. For MAML, this was done using a 10-shot to 24-task ratio with a three query outer update.

Balancing shot and task numbers is a critical challenge in FSL. Excessive shots can lead to overfitting, reducing generalization capacity. Conversely, too many tasks with insufficient shots can result in inadequate feature learning. While empirical studies suggest that prioritizing tasks over shots improves generalization, an optimal balance remains task-dependent. Our experimental design aimed to identify the most effective shot-task ratio for tooth mark classification within a meta-learning framework. We took after previous experimentation conducted to select balanced shot-task ratios during taxonomic classification using images of the occlusal surfaces of bovid teeth (in preparation). Earlier, before deciding on the two shot-task ratios described above, we had tried different combinations, from minimalistic 3-shot to 10-task ratio (which minimized overfitting during training, but decreased resolution and accuracy during classification of the hold-out testing set) to 15-shot to 20-task ratio, which yielded the least efficient models. The low- and high-shot solutions occupied an intermediate position of all the other experimental combinations that we tried.

### 2.9. Replication

All the dataset (image bank for the four carnivores) and code is available in the Harvard Dataverse public repository: https://dataverse.harvard.edu/dataset.xhtml?persistentId=doi:10.7910/DVN/WTPFBX.

### 2.10. Application to archaeo-palaeontological bone surface modifications

**Figure 1.**
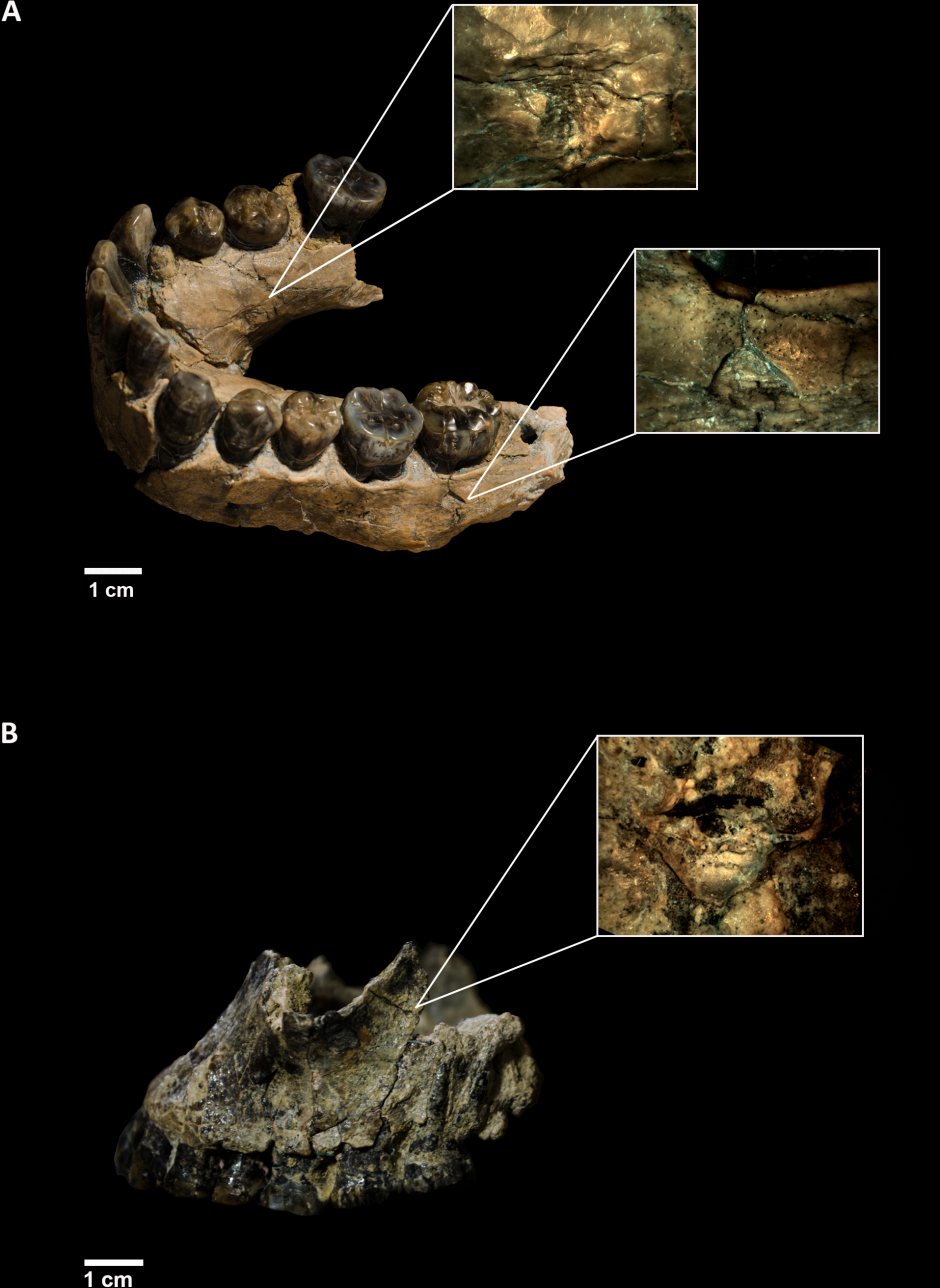
(A) OH7 mandible with the two tooth pits magnified. (B) OH65 maxilla with the tooth pit magnified. Tooth pits have been documented with a binocular Optika microscope and a 3 Mpx digital camera (OptiCamB3) with a magnification of ×10 in (A) and of ×30 in (B). Modified from Vegara-Riquelme *et al*. [34].

One way to comparatively assess the performance of different CV methods is to use models derived from DL and FSSL-MAML, not just on the same experimental assemblages but also in their classification of archaeo-palaeontological marks. Here, we will use a limited set of tooth marks discovered on two early *Homo habilis* specimens (OH7 and OH65) from Olduvai Gorge (Tanzania) (figure 1), which were previously classified as made by leopards, using a majority-voting approach to several DL models (ResNet50, DenseNet201 and VGG19) [34]. The classification method used here will consist of a weighted ensemble learning approach. For this purpose, we will use the five TL models described above through an FSSL architecture and four models (excluding EfficientNet V2L, due to computation requirements higher than the workstation used) for the MAML architecture. In order to make the ensemble learning more consistent, a weighted method will be implemented, consisting of considering each model’s specific weight as reflected by its accuracy in the classification of the testing sets. For a description of the Olduvai BSM and their location on the *H. habilis* fossils, we refer to [34].

We will consider only those BSM classified consistently both by the FSSL ensemble model and the MAML ensemble model (with probabilities greater than 70%) as reliable. Lower probabilities of individual BSM or lack of convergence between different methods will be taken as ‘interpretation with caution’, although we will still place reliance on the classifications derived from the ensemble learning methods, since it will be closest to the all-model convergence.

## 3. Results

### 3.1. Classification of the experimental set

#### 3.1.1. Few-shot supervised learning

The ResNet50 model that resulted with the highest accuracy was a combination of simple MAML architecture (using a 5-shot to 40-task ratio) with an Adam optimizer, which yielded an accuracy of 82% and a macro-average F1 score of 79%, with individual F1 scores ranging from 65% (crocodiles) to 88% (leopards) (tables 2 and 3). Hyenas, leopards and lions have F1 values greater than 80%, with leopard as the best score (88%), even though the hyena’s recall is the highest (91%) (table 3).

**Table 2.** Distribution of parameters (architecture type, optimizer and shot-task ratio) and performance metrics (accuracy, loss and macro-average F1 score) on the testing sets for each of the five TL FSSL models.

model	architecture	optimizer	shot	task	accuracy	loss	F1 score
ResNet50	simple	Adam	5	40	82	1.047	0.79
ResNet152	simple	Adagrad	5	40	83.59	0.452	0.83
Xception	simple	Adagrad	5	40	81.54	0.490	0.80
EfficientNet V2L	simple	Adam	5	40	81.03	0.713	0.79
DenseNet201	simple	Adam	5	40	82.56	0.534	0.81

**Table 3.** Classification metrics (precision, recall, F1 score) of the five FSSL models on the carnivore dataset.

ResNet50					
		precision	recall	F1 score	support
	crocodile	0.85	0.52	0.65	21
	hyena	0.72	0.91	0.8	54
	leopard	0.94	0.82	0.88	82
	lion	0.77	0.87	0.81	38
accuracy				0.82	195
macro-average		0.82	0.78	0.79	195
weighted average		0.84	0.82	0.82	195

ResNet152					
		precision	recall	F1 score	support
	crocodile	0.85	0.81	0.83	21
	hyena	0.77	0.89	0.83	54
	leopard	0.92	0.79	0.85	82
	lion	0.79	0.87	0.82	38
accuracy				0.84	195
macro-average		0.83	0.84	0.83	195
weighted average		0.84	0.84	0.84	195

Xception					
		precision	recall	F1 score	support
	crocodile	0.78	0.67	0.72	21
	hyena	0.8	0.87	0.83	54
	leopard	0.89	0.79	0.84	82
	lion	0.73	0.87	0.8	38
accuracy				0.82	195
macro-average		0.8	0.8	0.8	195
weighted average		0.82	0.82	0.82	195

EfficientNet V2L					
		precision	recall	F1 score	support
	crocodile	0.76	0.62	0.68	21
	hyena	0.76	0.89	0.82	54
	leopard	0.87	0.8	0.84	82
	lion	0.79	0.82	0.81	38
accuracy				0.81	195
macro-avg		0.8	0.78	0.79	195
weighted avg		0.81	0.81	0.81	195

DenseNet201					
		precision	recall	F1 score	support
	crocodile	0.79	0.71	0.75	21
	hyena	0.77	0.87	0.82	54
	leopard	0.91	0.82	0.86	82
	lion	0.78	0.84	0.81	38
accuracy				0.83	195
macro-average		0.81	0.81	0.81	195
weighted average		0.83	0.83	0.83	195

These results were improved by the ResNet152 TL model. In this case, the most accurate model (accuracy = 83.59) also yielded the highest macro-average F1 score of all the models and architectures in this study (83%). This was obtained by combining a simple FSSL architecture (using a 5-shot to 40-task ratio) with an Adagrad optimizer. Individual carnivore F1 scores ranged from 82% (lions) to 85% (leopards) (tables 2 and 3). Surprisingly, the crocodile subsample (which is the smallest on the four-carnivore set) yielded a F1 score of 83%. Crocodiles had never been classified as successfully before [27]. Even their recall was surprisingly high (81%). A slightly lower result was obtained with the Xception TL model. It yielded an accuracy of 81.54%, with a macro-average F1 score of 80%. The model was obtained using a simple FSSL architecture and an Adagrad optimizer (using a 5-shot to 40-task ratio). The individual carnivore F1 scores ranged from 72% (crocodiles) to 84% (leopards) (tables 2 and 3). The highest recall values were documented in hyenas and lions (table 3).

EfficientNet V2L also produced its best version using a combination of FSSL simple architecture and a 5-shot to 40-task ratio. With an accuracy of 81%, its macro-average F1 score was 79%, the lowest one (together with ResNet50) of the whole set, despite being by far the most complex and computationally expensive model. All carnivore taxa, except crocodiles, show F1 scores greater than 80%. In contrast with ResNet50, the crocodile F1 score is less than 70%. This renders the model the least adequate of the five models.

The last model, DenseNet201, also achieved high accuracy (82.56%), but it is not the best model, because its F1 score (81%) is slightly lower than that yielded by ResNet152 (83%). The model was obtained using the simple architecture (with a 5-shot and 40-task format) and using Adam as an optimizer (table 2). This model also produced balanced inter-taxonomic F1 scores, with only one of them being less than 80% (crocodiles = 75%) (table 3). As in some of the previous models, the highest recall was documented in hyenas and lions, even though the highest F1 score was observed in leopards (table 3).

It is interesting to note that in every single model, the lowest F1 score appeared in the crocodile subsample (which was expected given its smaller size) and the biggest F1 score was reported for leopards (which are the biggest sample size too). The ranking of F1 scores and recall values differed in every model according to carnivore, which renders the combination of these models in an ensemble learning optimal, because each of them has the potential to excel at classifying most efficiently a different type of carnivore. It is also interesting to note that the simple FSSL architecture produced better results than the highly regularized more complex architecture, indicating that the essential features separating carnivore taxa could be efficiently learnt with the simple FSSL structure.

#### 3.1.2. Model-agnostic meta-learning

The ResNet50 model achieved its highest performance when combined with a simple MAML architecture (5-shot to 40-task ratio) and optimized with Adam. This configuration yielded an overall accuracy of 85% and a macro-average F1 score of 84%**,** with class-specific F1 values ranging from 81% (crocodiles) to 88% (leopards) (tables 4 and 5). All carnivores attained F1 scores above 80%, with leopards exhibiting the highest score (88%), despite lions showing the greatest recall (97%) (table 3). These results surpass those obtained with FSSL.

**Table 4.** Distribution of parameters (architecture type, optimizer and shot-task ratio) and performance metrics (accuracy, loss and macro-average F1 score) on the testing sets for each of the four TL MAML models.

model	architecture	optimizer	shot	task	accuracy	loss	F1 score
ResNet50	simple	Adam	5	40	85	0.622	0.84
ResNet152	simple	Adam	5	40	82.56	0.744	0.82
Xception	simple	Adam	5	40	85.13	0.596	0.84
DenseNet201	simple	Adam	5	40	84.62	0.592	0.85

**Table 5.** Classification metrics (precision, recall and F1 score) of the four MAML models on the carnivore dataset.

ResNet50					
		precision	recall	F1 score	support
	crocodile	0.94	0.71	0.81	21
	hyena	0.85	0.81	0.83	54
	leopard	0.91	0.85	0.88	82
	lion	0.74	0.97	0.84	38
accuracy				0.85	195
macro-average		0.86	0.84	0.84	195
weighted average		0.86	0.85	0.85	195

ResNet152					
		precision	recall	F1 score	support
	crocodile	1	0.71	0.83	21
	hyena	0.78	0.83	0.8	54
	leopard	0.87	0.83	0.85	82
	lion	0.75	0.87	0.8	38
accuracy				0.83	195
macro-average		0.85	0.81	0.82	195
weighted average		0.84	0.83	0.83	195

Xception					
		precision	recall	F1 score	support
	crocodile	0.89	0.76	0.82	21
	hyena	0.79	0.89	0.83	54
	leopard	0.87	0.89	0.88	82
	lion	0.91	0.76	0.83	38
accuracy				0.85	195
macro-average		0.86	0.83	0.84	195
weighted average		0.86	0.85	0.85	195

DenseNet201					
		precision	recall	F1 score	support
	crocodile	0.94	0.71	0.81	21
	hyena	0.75	0.94	0.84	54
	leopard	0.92	0.84	0.88	82
	lion	0.83	0.79	0.81	38
accuracy				0.85	195
macro-average		0.86	0.82	0.83	195
weighted average		0.86	0.85	0.85	195

The ResNet152 model reached a maximum accuracy of 82%**,** producing a macro-average F1 score of 82% under the same MAML configuration. Class-specific F1 values ranged from 80 (lions and hyenas) to 85% (leopards), while the smallest subsample (crocodiles) yielded an unexpectedly strong score of 83% (tables 4 and 5). These outcomes are consistent with those obtained using FSSL.

The Xception TL model slightly outperformed ResNet152, achieving an accuracy of 85.13% and a macro-average F1 score of 82%. Under the same MAML framework, F1 scores ranged from 82% (crocodiles) to 88% (leopards) (tables 4 and 5). These results also exceeded those of FSSL.

The DenseNet201 model achieved one of the best performances overall, with an accuracy of 84.62% and the highest macro-average F1 score (85%) across all MAML (and FSSL) models tested. This model produced balanced interspecific F1 scores, ranging from 81% (crocodiles and lions) to 88% (leopards) (tables 4 and 5).

The MAML version of the EfficientNetV2L model was not included, because its computation requirements exceeded the memory of the workstation used for this study. Across all architectures, the ranking of F1 scores and recall values varied among carnivores, consistent with the pattern observed in FSSL models. This variability indicates that an ensemble learning approach would be optimal, as different models demonstrate strengths in classifying specific taxa.

### 3.2. Classification of the archaeo-palaeontological set

The use of the weighted ensemble FSSL model (including all the five TL architectures), classified the two OH7 marks as leopard-made (with probabilities ranging between 88 and 100%) and the OH65 tooth pit also as leopard-made but with a lower probability (44%) (table 6). This indicates that the leopard agency in OH65, while potentially reliable because of the convergent ensemble method embodying the five models, is not as reliable as that inferred for the other OH7 tooth marks, which display substantially higher probabilities (table 6). When using MAML as a contrasting method, the results are supportive of the FSSL results. Both of the OH7 tooth marks are classified as leopard-made (with probabilities of 93.3–99.8%), whereas the OH65 tooth mark is classified as made by a leopard but with a probability of 53.3%, which although substantially higher than the probabilities of the other carnivores, is still less than 70%, lower than the threshold that was set as reliable. The leopard agency for OH65 must, therefore, be taken with caution.

**Table 6.** Classification of the tooth marks identified on OH7 and OH65, comparing the ResNet50, DenseNet201 and VGG19 DL models [34], and the FSSL-MAML ensemble models from this study. Tooth marks 1 and 2 are both in the mandible of OH7, and mark 1′ is on OH65 maxilla. ID mark = Identification mark. Notice how the DL models were trained on five carnivores and the MAML models were trained on just the four African ones. Bold entries indicate the probability of the selected agent.

mark	ID mark	model	carnivores				
			crocodile	hyena	leopard	lion	wolf
**1**	**OH7 + 30 + 1**						
		DL ResNet50	0.006	0.007	**0.97**	0.003	0.02
		DL DenseNet201	0	0	**1**	0	0
		DL VGG19	0	0.0007	**1**	0	0
		FSSL ensemble	0	0	**1**	0	—
		MAML Ensemble	0	0	**0.99**	0.01	—
**2**	**OH7 + 15 + 2B**						
		DL ResNet50	0.02	0.002	**0.95**	0.02	0.005
		DL DenseNet201	0.0002	0.0006	**0.99**	0.005	0
		DL VGG19	0.001	**0.53**	0.46	0.0009	0
		FSSL ensemble	0.11	0.003	**0.88**	0.013	—
		MAML ensemble	0.02	0.02	**0.93**	0.033	—
**1´**	**OH65 + 45 + 1**						
		DL ResNet50	0.04	0.08	0.13	0.0006	**0.75**
		DL DenseNet201	0.002	0.02	**0.95**	0.02	0.002
		DL VGG19	0.06	0.44	**0.46**	0.03	0.007
		FSSL ensemble	0.43	0.12	**0.44**	0.011	—
		MAML ensemble	0.23	0.22	**0.53**	0.023	—

It should be remarked that the DL models compiled in table 6 (ResNet50, DenseNet201 and VGG19) were trained using five carnivore taxa [34] and the current FSSL-MAML models were trained using only the four African carnivores, since their application will be made on archaeo-palaeontological African faunal assemblages where wolves never were a potential intervening agent. Despite having been trained on different numbers of carnivores, it is interesting to note the convergence of agency determination as well as similar probabilities (with the exception of OH65) for the selected agent when comparing the DL and FSSL-MAML models.

## 4. Discussion

Previous modelling using DL methods yielded highly variable performance of the models used, ranging from 88% of accuracy (ResNet50; but with macro-average F1 score of 81) to 80% (EfficientNet B7; but with macro-average F1 score of 70) [21]. In both, the smallest taxon F1 score was for crocodiles (ranging from 40 to 60). Here, slightly lower accuracy scores were obtained, very probably because of the introduction of colour augmentation, levelling potential colouring differences that might have existed in the previous DL analyses according to taxon. In contrast to DL, the FSSL-MAML analyses presented here show a more homogeneous range of results, spanning from 83.56% accuracy (ResNet152) to 81.54% (Xception) for FSSL (table 2) and from 82.56% (ResNet152) to 85.13% (Xception) for MAML (table 4). The major improvement with the FSSL-MAML methods is that there is a significant increase in F1 scores (tables 3 and 5). For example, in the case of FSSL, there is a 13 full point increase of macro-average F1 values for all carnivores compared with the best previous DL model but a full 23 point increase in the same values when comparing the worst classified crocodile subsample to be best MAML model [21]. ResNet152 turned out to be the best performing FSSL model (83.56% of accuracy), with an equally high macro-average F1 score (83%). This resulted from the high taxon-specific F1 score ranges (crocodiles = 78, hyenas = 81, leopards = 82 and lions = 85) (table 3). This was improved with the application of MAML. In this case, Xception was the most accurate model (85.13%), with the following taxon-specific F1 scores (crocodiles = 82, hyenas = 83, leopards = 88 and lions = 83) (table 5). This is by far the best CV model produced to date to classify tooth marks from these four carnivore types.

Beyond its relevance to faunal assemblages, taxon-specific identification of carnivore activity has profound implications for reconstructing hominin evolutionary history. It has been argued that for a substantial portion of early hominin evolution—particularly during the australopithecine phase—hominins occupied a prey niche rather than a predatory one. The fossil record contains significant evidence of carnivore activity on hominin remains, suggesting a persistent predation risk. Felids, hyenids and crocodiles have all been presumably implicated as hominin predators, with direct evidence including tooth mark modifications on early hominin fossils. For example, crocodilian bite marks have been identified on *H. habilis* paratype fossils, highlighting their role as significant predators in aquatic environments [8,10]. Similarly, felid and hyenid modifications on hominin remains reinforce the idea that hominins were frequently subject to predation [26,36].

This perspective is critical for testing the ‘shift in the balance of power’ hypothesis, which postulates that hominins transitioned from being primarily prey to dominant hunters [37]. Determining whether hominins were consistently preyed upon and, if so, when and which hominin species experienced a trophic shift are major unresolved questions in human evolutionary studies. Addressing these issues requires a methodological framework capable of differentiating between specific carnivore agents rather than relying on broad categorizations. Likewise, the identification of taxon-specific carnivore agency is important for archaeological reconstructions of hominin–carnivore interactions. Understanding which carnivores were responsible for bone modifications informs interpretations of hominin behavioural strategies, particularly their potential for scavenging from felid kills [3]. For instance, the hypothesis that early hominins opportunistically exploited resources from felid kills relies on distinguishing between the primary access of lions to medium-sized carcasses and that of leopards to smaller carcasses [4,38]. In contrast, an alternative scenario—where hominins were the primary agents of carcass exploitation, followed by secondary intervention from durophagous carnivores such as hyenas—requires precise identification of hyena-induced modifications on bones within early archaeofaunal assemblages [7].

The models presented in this study indicate significant progress toward achieving the resolution necessary for this analysis. Specifically, their application to the selected set of BSM associated with two *H. habilis* specimens from Olduvai Gorge (OH7 and OH65) suggests that both individuals were preyed upon by leopards. These findings align with those obtained using DL models applied to the same marks [34].

The convergence of results across these methodologies is particularly significant, as it not only reinforces the identification of leopards as the modifying agents of the bone marks but also substantiates the methodological validity of both approaches. The DL models employed in previous studies were trained using a training–validation split, enabling them to learn predictive patterns from the available datasets. However, the absence of a separate hold-out testing set was cited as a limitation, raising concerns about the generalization potential of these models [39]. In response to this critique, subsequent research argued that DL models applied to BSM classification are constrained by the limited availability of training data, necessitating a trade-off between model learning and uncertainty in generalization [27]. The replication of results between the DL models and the meta-learning (FSSL-MAML) models presented in this study—along with the similarity in classification probabilities—demonstrates that the DL models were not overfitted to the validation set. Furthermore, these findings confirm that DL models exhibit generalization capabilities comparable to those of the FSSL-MAML models, validating their effectiveness in classifying modifying agents among the four carnivore taxa for which all models have been trained.

## 5. Conclusions

Simple FSSL-MAML architectures and low shot to task ratios yielded the most efficient models to date to differentiate tooth marks from four types of important African carnivores (crocodiles, leopards, hyenas and lions). The most accurate and balanced model (with 85.13% of accuracy and 84% of F1 macro-average score), provides precision-recall balanced metric (F1 score) for the smallest taxon-specific sample (crocodile) of 82% and for the other taxa greater than 80% (lion = 83%; hyena = 83%, leopard = 88%). This places substantial reliability in the classification of carnivore-made BSM.

However, as explained in depth by Domínguez-Rodrigo *et al*. [20,27], such confidence is only heuristically justified if the comparative analogical frameworks are equitable; that is, if when applied to palaeobiological BSM, the preservation of those is unaffected by conspicuous biostratinomic and diagenetic processes. If impacted by diagenetic factors, this method may be unreliable. Therefore, the method is objectively efficient, but its reliability depends on how it is applied by taphonomists to the fossil record. We acknowledge the limitations inherent in extrapolating experimental models to palaeobiological contexts. Controlled experiments, such as those reported in Domínguez-Rodrigo *et al.* [21] and Vegara-Riquelme *et al.* [34], are designed to generate tooth marks under conditions in which variables such as the carnivore agent, the skeletal elements and the feeding sequence are carefully monitored. These models provide a critical framework for identifying diagnostic traits, but we recognize that prehistoric/fossil assemblages often present additional challenges. Bone surfaces may be affected by overlapping traces from multiple taxa, differential preservation or taphonomic overprinting that can obscure morphological criteria. As highlighted in the literature, these factors complicate taxonomic attribution of marks in natural contexts and demand a cautious interpretive approach. Nevertheless, the experimental framework employed here is robust in that it establishes replicable, well-defined referential patterns that allow for consistent recognition of tooth mark morphology. By grounding field identifications in such controlled referentials, we reduce subjectivity and increase the reliability of our interpretations, even while acknowledging that contextual complexity in the field requires careful integration of multiple lines of evidence.

The application of the new models to the tooth marks found on two emblematic *H. habilis* fossils (OH7 and OH65) revalidates recent interpretations derived from the use of DL models [34] and certifies that felids (namely, leopards) continued to be a hazard for hominins well into the early *Homo* evolutionary stage.

## Data Availability

Data and code are available in the public repository described in the manuscript [40].
